# How automation level influences moral decisions of humans collaborating with industrial robots in different scenarios

**DOI:** 10.3389/fpsyg.2023.1107306

**Published:** 2023-03-09

**Authors:** Anne Eich, Anja Klichowicz, Franziska Bocklisch

**Affiliations:** Research Group “Human-Cyber-Physical Systems”, Institute of Materials Science and Engineering, Materials and Surface Engineering Group, Chemnitz University of Technology, Chemnitz, Germany

**Keywords:** human-robot-collaboration, human-machine-teaming, level of automation, moral dilemma, decision-making

## Abstract

**Introduction:**

Digitalization in intelligent manufacturing leads to the development of Industry 4.0/5.0 and human-cyber-physical systems. As many production technologies rely on teaming of human workers and intelligent cyber-physical systems such as industrial robots, human-robot collaboration is an intensively investigated topic in this transdisciplinary research area. To design industrial robots in a human-centered way, psychological knowledge concerning judgment and decision-making needs to be gained and integrated.

**Method:**

This paper reports results from an experimental study (*N =* 222, 2 × 4 within-subjects design) using eight moral dilemmas framed in the context of human-robot-collaboration to examine the influence of spatial distance of an industrial robot and humans (no direct contact, different tasks vs. no direct contact, same task vs. handing-over contact, same task vs. direct contact, same task) on moral decisions. Additionally, the type of dilemma was varied, with every four dilemmas depicting a life-or-death and an injury scenario. Participants responded on a four-point-response scale which actions they would take indicating deontological or utilitarian moral decision-making.

**Results:**

Results show a large effect of the proximity of the cooperation between robots and humans. The closer the collaboration the more a human tends to choose utilitarian moral choices.

**Discussion:**

It is argued that this effect might stem from an adaptation of human rationality to the robot or overreliance and shift of responsibility to the robot team partner.

## 1. Introduction

### 1.1. Background and motivation

**Figure 1:**
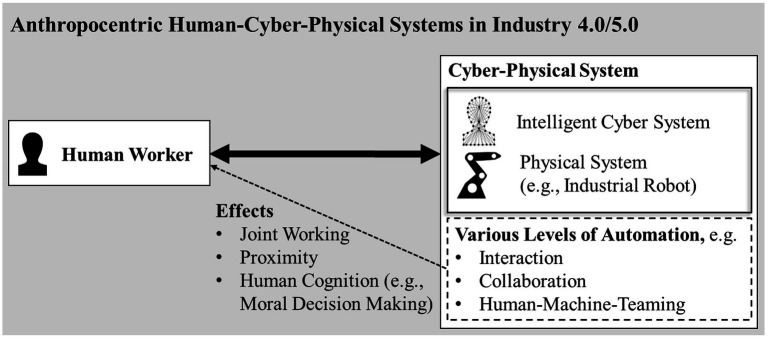
Anthropocentric human-cyber-physical systems in industry 4.0/5.0, exemplar level of automation and selected effects relevant for the presented study.

Recent development toward Industry 4.0/5.0 requires a strict anthropocentric perspective and clear transdisciplinary concepts to foster a good, human-centered, and entirely sustainable technical evolution (Rauch et al., 2020; Xu et al., 2021; Bocklisch et al., 2022). One promising conceptualization is human-cyber-physical systems (HCPS; Madni and Madni, 2018; Bocklisch et al., 2022) as it considers the human as a central part of the system and renders possible drawing conclusions regarding upcoming research topics and effects on the human worker. Figure 1 shows the basic parts and relations in HCPS, various possible levels of automation (including collaboration and human-machine-teaming), and a selection of effects or consequences that might arise. Although very simplified, it summarizes and systematizes the main background and motivation of the present study.

Nowadays, robots have become indispensable in intelligent manufacturing. They represent a crucial factor to keep up economically on the international market (Heyer, 2010; Villani et al., 2018; Bröhl et al., 2019). Industrial robots partly replace humans performing monotone, potentially dangerous, or harmful tasks (Robla-Gómez et al., 2017), such as welding, sorting, or stacking (Heyer, 2010). Most industrial robots operate fixed in a cell, spatially separated from human workers, and perform a limited set of actions (Heyer, 2010). This spatial separation guarantees human safety (Robla-Gómez et al., 2017) as collision with heavy robots is life-threatening. Nevertheless, progress in information and communication technology, intelligent sensors, and artificial intelligence lead to next-generation robots, which show more enhanced collaboration abilities. Consequently, humans and robots may share a workspace in closer proximity leading to higher flexibility, individuality, shorter production times, and more sustainable use of resources (Çürüklü et al., 2010; Ajoudani et al., 2018; Kadir et al., 2018; Bröhl et al., 2019; Yuschenko, 2020). Current trends do not see a focus on full automation of production processes but on collaboration and teaming between humans and machines (Madni and Madni, 2018; Bocklisch et al., 2022) to combine different strengths in a complementary way, for instance, human cognitive flexibility and robots preciseness (Villani et al., 2018; Weitschat, 2018; Weiss et al., 2021). Hence, the effects of closer physical proximity and different levels of automation on the human worker need to be studied carefully and with special regard to psychological processes including judgment and (moral) decision-making (see Figure 1 and below).

### 1.2. Levels of human-robot interaction and consequences for human decision-making

Bdiwi et al. (2017) distinguish four levels of interaction between humans and robots, which differ in human-robot proximity and autonomy in joint working tasks as follows:

Level (1): Spatially separated workplaces divided into two zones, with the robot remaining in one zone and the human being able to switch between zones. If the human enters the robot’s zone, the robot immediately stops its movements to guarantee worker safety.

Level (2): Spatially separated workplaces and an additional third “cooperation” zone. Humans and robots work on the same task but without physical contact. The robot can move into the cooperation zone and get closer to the human to help him solve the task. For safety purposes, the robot’s speed slows down within the cooperation zone.

Level (3): Shared workspace and shared task including direct handing-over of a building component or tools between the robot and human. The robot can react to the movements of the human and adjust its speed accordingly.

Level (4): Robots and humans work in a shared workspace on a joint task with direct physical contact. Here, both are equally responsible for the execution and task performance.

Although robot autonomy increases with higher levels of interaction, robots do not have the ability to take “responsibility” deliberately as the human worker does. Hence, the ultimate responsibility for decisions and actions rests with the human also in morally difficult situations (Çürüklü et al., 2010). Nevertheless, it is possible that the human perception of control over the joint working situation is shifting with higher levels of automation. On this account, humans might become less willing to take responsibility the closer the collaboration with robots becomes. Overreliance effects and shifts in responsibility a well-known side effect (Lee and Moray, 1992) which may as well result in decreased emotional involvement of the human (Weiss et al., 2021). Depending on the autonomy and perceived “intelligence” of the robot, the human worker may tend to see the robot as an equal teammate rather than a tool. This might affect human (moral) decision-making. As one undesired consequence of human-machine teaming, humans might adapt themselves to the “unemotional” and rational technical sphere resulting in changed moral decisions (Çürüklü et al., 2010). Furthermore, humans could be more willing to take deliberate, utilitarian actions to behave in a predictable manner and avoid safety hazards (Gerst, 2020). Although technical developments offer many new possibilities and robots are considered safe, the remaining risks in human-robot collaboration need to focus on human moral decisions as well. This becomes increasingly crucial because robots are supposed to become more present in everyday life as their fields of application are expanding, for instance, to service and health care (Herrmann and Melhuish, 2010). The influence of perceived proximity and level of automation on moral decisions has not yet been studied in the context of human-robot-collaboration. As a profound psychological understanding of the consequences of human-robot-teaming for human decisions is important to improve interaction, the recent study aims to address the research gap. In the following, we present a short summary of the recent state of research in decision-making and moral judgment relevant to the topic. Thereafter, we derive the research question and hypotheses.

### 1.3. Theories of judgment and decision-making and moral psychology

Judgment and decision-making have extensively been studied in cognitive psychology. As one example, the dual-process theory arose in the mid-1990s. According to this theory, two distinct cognitive systems are important for thinking or decision-making (Evans, 1984; Sloman, 1996; Pelaccia et al., 2011; Hayakawa et al., 2017): “System 1” and “System 2” (see Table 1).

**Table 1:** Relationship of (1) dual-process-theory and (3) moral decision-making and (3) hypothesized effects on **(A)** moral dilemma decisions and **(B)** associated automation level/proximity in human-robot-collaboration.

(1) Dual process theory		(2) Moral decision-making	(3) Hypothesized effects on	
			(a) Moral dilemma	(b) Automation level/proximity
System 1	Associative thinking fast intuitive	Deontological emotional context-independent	Life/death	Low
System 2	Deliberate thinking time-consuming rule-based	Utilitarian rational context-dependent	Injury	High

The “associative system” leads to faster, more intuitive processes with less cognitive expense and decisions based on habits, similarities, and continuity. Hence, it is rather inflexible and harder to control (Sloman, 1996). In contrast, the “deliberate system” goes together with slower, more time-consuming, and willful thinking processes (Sloman, 1996; Kahneman, 2003). Decisions are mostly based on the analysis of additional information, rules, and logic structures. As a result, the thinking processes are related to a much higher cognitive expenditure than those of system 1 (Sloman, 1996; Pelaccia et al., 2011). The dual-process theory is criticized from time to time, for instance, because it does not serve an evolutionary perspective on decision-making or it might be a too simple construct for the complexity of cognitive processing (Evans, 2011; Talat et al., 2017). Nevertheless, it is a good starting point for research and should serve as a motivation for further investigations regarding decision processes and cognitive development (Evans, 2011).

Current trends in psychological moral research use dual-process theory to explain moral decision-making and judgment. It is assumed that there are, equivalent to the two cognitive systems, two types of morals that are controlled by these systems (Hayakawa et al., 2017). In moral research, the automatic processes of system 1 are related to the term “deontology,” while the analytic processes of system 2 are described with the term “utilitarianism” (Greene et al., 2001; see Table 1). The deontological moral describes the case when the moral reasonableness of an action is dependent on how compatible it is with the prevailing moral norms and rules (Gawronski and Beer, 2016; Białek et al., 2019; Brouwer, 2021). The moral norms are not weighed consciously or on purpose, but they are used as a guideline subconsciously to lead to an intuitive decision based on continuity and habit. Therefore, emotional processes control deontological judgments and are correspondingly automatic, fast, and involuntarily. The utilitarian moral refers to actions that are dependent on their consequences. In this form of morality, the action is selected which results in the largest benefit for the largest group of people, in order to maximize advantages and usefulness. In contrast to deontology, utilitarianism is free of affective processes and characterized by a slower, laborious, and more analytic way of processing. This purposeful weighing of advantages and disadvantages requires abstract knowledge and practice (Gawronski and Beer, 2016; Čavar and Tytus, 2018; Muda et al., 2018; Brouwer, 2021). In conclusion, deontological moral means that a person tries to follow the current norms and rules when making a moral decision. Hence, deontology is independent of each situation. For example, regarding the deontological moral, it would be unacceptable to kill one person to save a group of other people because it would break the universal rule of not killing other people (Białek et al., 2019). On the contrary, utilitarian morality is dependent on the current situation because each situation requires a new rational weighing of costs and benefits. Following the example above, utilitarian morality accepts killing one person to save others. The one person, who dies, is an “acceptable” sacrifice to avoid greater harm. However, killing a person without any positive consequences for the greater good, would not be acceptable in the eyes of the utilitarian moral, emphasizing the importance of the situation when reaching decisions (^12,8^). Greene et al. (2001) propose that emotional response and, therefore, a deontological decision is more likely when a person is confronted with a personal dilemma, meaning the person is more involved in the dilemma and must participate actively in the situation, like pushing a person in front of a train in the footbridge dilemma. Hence, the emotional reaction triggered by a dilemma is the crucial factor for a person either deciding in a deontological or utilitarian manner. However, there are voices claiming it remains unclear what leads a person to take a deontological or utilitarian decision, stating that there can be more factors than emotionality in decision-making, like the possible outcomes, neural responses, or personality (e.g., Gawronski and Beer, 2016).

To operationalize moral decisions, participants are often confronted with decision tasks like the footbridge dilemma (Thomson, 1976). This philosophical dilemma is one of many depicting a hypothetical situation in which a person must choose between various numbers of death victims (Paxton et al., 2012). Often, these dilemmas display theoretical situations which are unlikely to occur in a usual work environment of an average person, for instance, situations of war. Hence, it is important to consider more lifelike dilemmas to investigate moral behavior in rather realistic environments, such as in the workplace and questions of injury instead of death (Geipel et al., 2015). By investigating realistic moral decisions beyond life-or-death scenarios, a person’s choice could vary depending on the type of scenario they are confronted with. For example, the terror management theory (Greenberg et al., 1997) proposes that the confrontation with death frightens people resulting in control strategies coming from their own culture and symbols for stability and security. Thus, people could possibly tend to decide more deontological when facing a situation where life and death are at stake because they might subconsciously refer to a social or religious rule to not kill others, and therefore, a singular person should not be sacrificed actively to save a group of people. A choice regarding an injury could be different from a life-or-death situation because one life cannot be weighed against another valuable life (e.g., see prospect theory, Kahneman and Tversky, 2013). Furthermore, the question of injury in workplace environments is a very realistic one, for instance, when human-machine-teaming in human-cyber-physical systems (Bocklisch et al., 2022) and human-robot-collaboration scenarios are considered.

### 1.4. Aim and hypotheses of the present study

This study aims to investigate the influence of interaction levels (Bdiwi et al., 2017), operationalized by spatial distance and degree of automation, on human moral decisions in a fictive production workplace scenario. Thus, the dependent variable is the moral decision (deontological vs. utilitarian), which is assumed to be influenced by the kind of dilemma (life/death vs. injury dilemma) and the interaction levels (see Table 1). Considering these factors, we hypothesize that:

1. Life/death dilemmas result in a more deontological decision than injury dilemmas
2. the higher the interaction level, that is, the proximity between human and robot, the more utilitarian the choices are.

## 2. Method

### 2.1. Participants

In total, 265 people took part and 223 participants completed the study. One outlier had to be excluded, leading to a valid sample size of *N* = 222. The recruitment of the test persons took place on the one hand *via* the internal mail distribution list of the University, on the other hand, the study was distributed *via* the online platform SurveyCircle. Based on this method of recruitment, it can be assumed that this is mainly a student sample. All participants took part in the study voluntarily. From the valid sample, 56 participants were male (75% female) and the average age was 26.31 years (SD = 9.32). Most of the participants stated a general university entrance qualification as their highest degree (53%). All procedures performed in this study involving human participants were in accordance with the ethical standards of the institutional research committee (institutional ethics approval number #101520517).

### 2.2. Design

The study was carried out as an online survey in a 2×4 within-subjects design, with two types of dilemmas (life/death vs. injury dilemma) and four human-robot interaction levels (Bdiwi et al., 2017) as independent variables. Each participant responded to the eight dilemmas within the context of human-robot collaboration.

### 2.3. Material

The dilemmas were developed for this study, based on the Footbridge Dilemma (Thomson, 1976). Altogether, there were two scenarios for each level of human-robot interaction according to Bdiwi et al. (2017). To construct the dilemmas accordingly, the levels of interaction by Bdiwi et al. (2017) were equated to the spatial distance between the human worker and the robot as well as the automation of the robot, varying in (1) the task and (2) the workspace. Therefore, in dilemmas of level 1, the human and the robot are working on separate tasks in separate locations, whereas in level 2 the human and the robot share a task in a cooperative working space with the robot assisting the human by holding building components in place while the human can assemble them. The dilemmas in level 3 described a situation where the human and the robot share a task as well as a working space, having the ability to interact by handing over processes. Lastly, the dilemmas of level 4 comprise the human and the robot sharing a task as well as a working space where the robot holds a building component, and the human worker brings it to its final position using his own physical force.

There were two dilemmas per interaction level, with each dilemma representing a life/death dilemma and one an injury dilemma. In the life/death dilemmas, the participant had to weigh the number of fatalities and decide how many people would be sacrificed. This type of dilemma was very similar to the original Footbridge dilemma (Thomson, 1976). In contrast, the injury dilemmas required participants to balance the risks and injuries of colleagues. This difference from the original Footbridge Dilemma was chosen to create a more real-life situation. The dilemmas were introduced with the help of the following cover story (example dilemma level 1, life/death scenario; other dilemmas see Appendix A; Table 1).

*Together with your human colleagues and a robot you are working on separate tasks in individually allocated areas in a working space without fences. Due to an error, the robot leaves its allocated area moving toward five of your colleagues at a life-threatening speed. Your own body is too light to stop the force of the robot, however, if you would push another taller colleague into the robot’s path, the robot would be held back by your colleague’s bulkier body. This colleague would most definitely die. Would you push your colleague into the robot’s path in order to save the five other colleagues?*

### 2.4. Procedure

Before participating in the study, participants were informed about the potentially stressful content of the study and asked for a declaration of consent. The dilemmas were presented with increasing levels of interaction according to Bdiwi et al. (2017). The participant’s task was to indicate their action tendency on a scale from 1 (“yes”) to 4 (“no”) after each dilemma. Depending on each dilemma, the answers either represented a utilitarian or a deontological answer. In the preceding example (see Material above), “yes” indicates a utilitarian and “no” a deontological answer. This continuous scale was chosen to assess the uncertainty or hesitation of the participants (Čavar and Tytus, 2018). In addition, this scale had no center category to make the decision more realistic, as a person would also not have the possibility of a neutral position when faced with this situation in real life.

After responding to all dilemmas, participants rated their perceived confidence with their given answers (see Geipel et al., 2015) on a five-point Likert scale (1 = “very uncertain”; 5 = “very certain”).

## 3. Results

**Figure 2:**
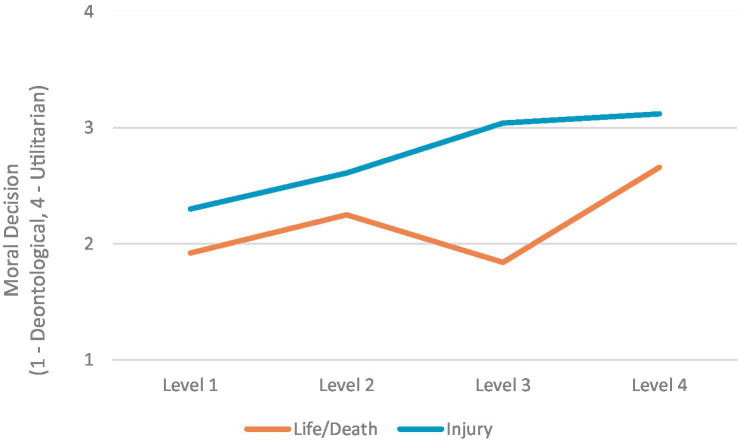
Moral decisions in different dilemma types and on different levels of automation.

A total of *N* = 222 completely answered questionnaires were included in the statistical analysis. During the study, the type of judgment (deontological vs. utilitarian) depending on the spatial distance of a robot to human co-workers and the type of dilemma was recorded (see Figure 2). Since depending on the dilemma “yes” and “no” could either stand for a utilitarian or a deontological decision, the answers were dummy coded. Therefore, a deontological judgment was given a numerical value of 1, while a numerical value of 4 represents a utilitarian judgment. Except five combinations, most dilemmas did not correlate with each other significantly (Appendix A; Table 2). Among the significant ones, for example, the highest correlations were found for scenarios level 1 life/death and level 3 life/death (*r* = 0.64) and between level 3 injury and level 4 injury (*r* = 0.51). A repeated-measures ANOVA was performed with the factor type of dilemma (life/death vs. injury) and interaction level (levels 1 to 4). We found a significant main effect on the moral decisions in different dilemma types (*F*(1, 220) = 150.51, *p* < 0.001, *η*_p_^2^ = 0.41). Further, there was also a significant main effect between the moral judgments in the different interaction levels with a large effect size (*F*(1, 220) = 65.86, *p* ≤ 0.001, *η*_p_^2^ = 0.23). Bonferroni pairwise comparisons revealed meaningful differences between all human-robot interaction levels (all *p*s < 0.001) except levels two and three (*p* = 1). We also found a significant interaction between the human-robot interaction level and the type of dilemma (*F*(1, 221) = 23.26, *p* = 0.001, *η*_p_^2^ = 0.95) although this was not included in our hypotheses.

## 4. Discussion

In this study, we explored the influence of the type of dilemma a person is confronted with and the perceived proximity of collaboration between humans and robots on moral decisions. It turns out that both factors have a large effect on moral judgment. In line with the first hypothesis, results show that moral decisions seem to be more utilitarian when a person is confronted with injury dilemmas than with life/death dilemmas, indicating a tendency to decide more rationally in non-life-threatening situations. Additionally, the second hypothesis could be confirmed as well: A smaller spatial separation between humans and robots leads to more utilitarian decisions. The fact that human-robot-interaction levels 2 and 3 do not differ significantly could result from their similarity. Maybe the difference was not as clear to the subjects. In addition, a significant interaction between the dilemma types and interaction levels could be found which was not hypothesized. This interaction stems from the fact that in level 3, the life/death dilemmas result in deontological answers compared to the injury dilemmas of the same level, which show rather utilitarian responses. Although the material was designed carefully, it cannot be ruled out completely that the statistical interaction between dilemma types and human-robot-interaction levels might have methodical reasons (just one dilemma for each combination). In future studies, more experimental trials per condition should be presented. Additionally, it should be considered that—on the one hand—level 1 and level 3 scenarios are considerably different concerning the perceived (physical) proximity of the robot (separate zones without contact vs. cooperation zone and direct physical handover situations). On the other hand, the robot’s failure mentioned in both scenarios is the same: An “erratically movement.” As this was not specified further, the error cannot be attributed to the logic of the working task itself and, therefore, may seem “random” or without causal explanation to the participants. Consequently, system 2 has no starting point for reflection. Therefore, things that happen more “accidentally” may trigger system 1, and hence, deontological decisions. In life/death scenarios 2 and 4, this is a bit different. Herein, the problem leading to the life-threatening course of action is more closely associated with the working movements of the robot (holding or lowering of heavy components). Hence, analytic thinking about causes of potential malfunctions might be more reasonable even if not this was not triggered consciously or forced by the experimental design. But it could be one explanation for the higher tendency toward utilitarian, analytic, and system 2-based moral judgments. Why this effect is not evident for injury dilemmas is not completely clear yet but may be due to the fact that injury vs. life/death results in such fundamental differences or consequences for human integrity and life. By tendency, this is seen in the correlation matrix results (only scenarios of the same type correlate significantly) and is in line with our first hypothesis (see above). As the material was carefully constructed and questions did not trigger thinking/not thinking about explanations of robots malfunctions, this issue should be studied again, for instance, by explicit manipulation. Furthermore, not only physical proximity is of relevance in HCPS and human-robot-teaming but perceived cognitive autonomy of the technical system might become increasingly important as well. As the cyber-system or “cognitive” system of the machine (see Figure 1) may have different levels of maturity and possibilities to act in an intelligent, context-specific, and adaptive way, (moral) decisions might be influenced through cognitive attributions.

Strengths of this study are (1) the high power due to 222 valid questionnaires, which is more than twice as many participants that would have been required for sufficient power, (2) the dilemmas presented were set in a tangible work context related to human-robot collaboration, which—to our knowledge—has not been done before. Further, (3) a new type of more lifelike dilemmas, the injury dilemmas, were introduced, which allow a new perspective on moral judgment.

Potential study limitations are: (1) that the sample might not be representative for an industrial work population because of a relatively young average age, as well as a high proportion of female participants and students. Additionally, the subjects mostly had higher levels of education. It has been shown, that attending a university, or a comparable educational institution has an important influence on the development of moral judgments, for example, due to the socialization processes (Rest, 1988). Thus, with a more representative sample, the results could differ from the present study, since people without a university background might have gone through a different development of their moral value system. Moreover, in future research, having a representative sample from the human-robot collaboration context could increase the realism and practical relevance of the results. Nevertheless, the sample and results are of considerable interest for basic research and will be extended to the applied field in a next step. In addition, (2) the internal consistency and the dilemma have not yet been proven sufficiently as they are newly constructed. Footbridge and Trolley dilemmas reliably measure the action tendency and therefore give an accurate insight to likely actions taken by subjects. Furthermore, they state a very good starting point for new research fields in moral decision-making. But the subject’s real intention for choosing a particular action cannot be evaluated only using footbridge and trolley dilemmas (Gawronski and Beer, 2016). There is yet no material developed that can measure the action tendency as well as the intention of the subjects reliably, leaving room for further investigations in this field of research. An additional limitation is that (3) the presentation order of the scenarios was fixed. Therefore, possible order effects cannot be evaluated. However, there is evidence that moral judgments are very consistent in humans (Helzer et al., 2016) and we do not think that presentation order impacted the results considerably. As no feedback was given to the participants, we do not expect learning or carry-over effects either. Nevertheless, in future studies and if more scenarios are presented, these factors need to be controlled adequately. A final possible limitation that we want to address is (4) the question of transferability. This means that subjects are often motivated to do their best in tests and may even intend to impress the examiner (Krebs et al., 1997). In addition, people often adapt their moral judgment to their audience (Carpendale and Krebs, 1992), so there is a risk that the behavioral tendencies expressed in this study do not correspond completely to actual behavior in a real situation. However, since this was an anonymous online study where no direct interaction with the experimenter took place, we are confident that we were able to keep this influence on a minimum. Future investigations could increase the closeness to real-world work situations by using video material or pictures or creating time pressure for the participants thereby also expanding the control of the involvement of participants. Furthermore, it can be of interest to make use of a between-subjects design in future research to grant higher controllability of the sample as well as to investigate whether the design has an influence on the results. As well, including further variables, such as experience or emotional intelligence, might also be interesting. To implement this, the use of a manipulation check of the task and the use of personality tests and appropriate measures, for example, emotional intelligence tests would be of interest.

In summary, we showed the importance of investigating moral judgments in the context of human-robot collaboration using new dilemmas, by creating a more lifelike test situation. The physical separation between humans and robots in workplaces continues to shrink, additionally, the operation area of robots is expanding, which might lead to the expectation that direct interaction between humans and robots will be an integral part of future society. Hence, it is of utmost importance to be aware of the impact of close human-machine collaboration and teaming on moral judgment. By understanding the process of moral decision-making in the context of human-robot collaboration, safety hazards can be minimized and, as a result, human-robot interaction can be improved. For this, it is necessary that there is further research in this area and that the results find their way into practice. This could be done, for example, by training and sensitizing human workers interacting with robots accordingly so that there is no shift in responsibility in dangerous situations.

## Data availability statement

The raw data supporting the conclusions of this article will be made available by the authors, without undue reservation.

## Ethics statement

The studies involving human participants were reviewed and approved by the Ethics Committee of Chemnitz University of Technology. The patients/participants provided their written informed consent to participate in this study.

## Author contributions

AE and FB developed the research question, theory, and experimental plan. AE and AK carried out the experiment. AE analyzed, interpreted, and discussed results and wrote the main parts of the manuscript. FB supervised the research project and contributed to manuscript finalization. All authors contributed to the article and approved the submitted version.

## Funding

The publication of this article was funded by the Chemnitz University of Technology.

## Conflict of interest

The authors declare that the research was conducted in the absence of any commercial or financial relationships that could be construed as a potential conflict of interest.

## Publisher’s note

All claims expressed in this article are solely those of the authors and do not necessarily represent those of their affiliated organizations, or those of the publisher, the editors and the reviewers. Any product that may be evaluated in this article, or claim that may be made by its manufacturer, is not guaranteed or endorsed by the publisher.
