# Supplementary material for: How automation level influences moral decisions of humans collaborating with industrial robots in different scenarios
Source: Front Psychol. 2023 Mar 9;14:1107306. doi: 10.3389/fpsyg.2023.1107306 (PMC10035336; doi:10.3389/fpsyg.2023.1107306)
Supplement: Supplementary file 1 [file Table_1.docx]

Supplementary Material to Manuscript Eich et al.:

How the Automation Level Influences the Moral Decisions of Humans Collaborating with Industrial Robots in Different Scenarios

**Appendix A: Table 1**

*Dilemmas overview*

| Interaction | Dilemma-Type | Dilemma |
| --- | --- | --- |
| Level 1 | Life/Death | Together with your human colleagues and a robot you are working on separate tasks in individually allocated areas in a working space without fences. Due to an error, the robot leaves its allocated area and it is moving towards five of your colleagues at a life-threatening speed. Your own body is too light to stop the force of the robot, however if you were to push another taller colleague into the robot’s path, the robot would be held back by your colleague’s bulkier body. This colleague would most definitely die. Would you push your colleague into the robot’s path so that the five other colleagues can be saved? |
|  | Injury | Together with your human colleagues and a robot you are working on separate tasks in individually allocated areas in a working space without fences. Due to an error, the robot leaves its allocated area and it is moving towards one of your colleagues at a life-threatening speed. If the robot were to hit this colleague, he would most definitely end up suffering from paraplegia. If you were to push the colleague at risk out of the path of the approaching robot, you could save him, however this would include the risk of him suffering from life-threatening head injuries. Would you push your colleague out of the robot’s path to avoid him from suffering paraplegia, although this entails the risk of him suffering from life-threatening head injuries? |
| Level 2 | Life/Death | Together with your human colleagues and a robot you are working on a joint task in a cooperative working space. The robot can hold construction components, whereas the human colleagues assemble these components. Due to an error, the robot begins moving erratically and there is a risk that it may throw one of the components it is holding towards a colleague. Most definitely, your colleague’s life would be endangered if he was hit. It would be possible to push your colleague out of the danger zone. However, this would result in a deadly hit of five other of your colleagues as the component’s force would not be slowed down by another body. Would you leave your colleague in the danger zone in order to save your five other colleagues? |
|  | Injury | Together with your human colleagues and a robot you are working on a joint task in a cooperative working space. The robot can hold construction components, whereas the human colleagues assemble these components. Due to an error, the robot begins moving erratically and there is a risk that it may drop one of the components it is holding onto one of your colleagues. Most definitely, your colleague would lose an arm if the component fell onto him. It would be possible to push your colleague out of the danger zone; however, this might cause him to suffer life-threatening head injuries. Would you push your colleague out of the path of the falling component in order to avoid the definite loss of his arm, despite there being the possibility of a possible life-threatening head injury? |
| Level 3 | Life/Death | Together with your human colleagues and a robot you are working on a joint task in a working space without fences. The robot has the ability to bring tools or construction components and to hand them to human coworkers. Due to an error, the robot begins moving erratically and there is a risk that the robot may hit five of your colleagues while doing a hand-over of a component with sharp edges, risking their lives. Your body is too light to stop the force of the robot. However, if you were to push another taller colleague into the path of the robot, the robot and the component with the sharp edges would be stopped. You colleague would most certainly die. Would you push your colleague into the path of the robot and the component with the sharp edges in order to save five others? |
|  | Injury | Together with your human colleagues and a robot you are working on a joint task in a cooperative working space in a working space without fences. The robot has the ability to bring tools or construction components and to hand them to human coworkers. Due to an error, the robot gets hold of the hand of one of your colleagues and does not let go of it. If you wait for the team that can carefully free the hand by unscrewing it, it is possible that your colleague’s hand can be sustained. In great pain your colleague pleas that you forcefully free his hand out of the robot’s grip, risking that he might lose his entire arm. Would you free the hand out of the robot’s grip by force in order to adhere to your colleague’s wish, although your colleague could lose his entire arm? |
| Level 4 | Life/Death | Together with your human colleagues and a robot you are working on a task in a working space without fences. The robot has the ability to bring heavy components and you have to put them into their final position by using your own physical powers. Due to an error, the robot lowers the heavy component too quickly and thus crushes one of your colleagues. You could pull your colleague out by using your own physical powers. However, this would mean that five other colleagues would sustain a deadly hit by this component, as they would not have sufficient time to evade. Would you not pull your colleague out and leave him under the component in order to save five other colleagues? |
|  | Injury | Together with your human colleagues and a robot you are working on a task in a working space without fences. The robot has the ability to bring heavy construction components and you have to put them into their final position by using your own physical powers. Due to an error, the robot lowers the heavy component too quickly and thus crushes one of your colleagues. By pushing a button, you could make the robot lift the component immediately, however the colleague would most definitely lose his leg. If you leave the component in its place and wait for the medical professionals, there is a chance that you can still save the leg. In great pain your colleague pleas that you lift the component. Would you let the robot lift the component in order to meet your colleagues’ wish, although he will most definitely lose his leg? |
