# Supplementary material for: How automation level influences moral decisions of humans collaborating with industrial robots in different scenarios
Source: Front Psychol. 2023 Mar 9;14:1107306. doi: 10.3389/fpsyg.2023.1107306 (PMC10035336; doi:10.3389/fpsyg.2023.1107306)
Supplement: Supplementary file 2 [file Table_2.docx]

Supplementary Material to Manuscript Eich et al.:

How the Automation Level Influences the Moral Decisions of Humans Collaborating with Industrial Robots in Different Scenarios

**Appendix A: Table 2**

Correlations Between Dilemma-Conditions

| Dilemma | Level 1, Life-Death | Level 1, Injury | Level 2, Life-Death | Level 2, Injury | Level 3, Life-Death | Level 3, Injury | Level 4, Life-Death |
| --- | --- | --- | --- | --- | --- | --- | --- |
| Level 1, Life-Death | - |  |  |  |  |  |  |
| Level 1, Injury | -.06 | - |  |  |  |  |  |
| Level 2, Life-Death | -.23* | .03 | - |  |  |  |  |
| Level 2, Injury | .03 | .43* | -.02 | - |  |  |  |
| Level 3, Life-Death | .64* | -.02 | -.29* | .07 | - |  |  |
| Level 3, Injury | -.11 | -.12 | .06 | .04 | .-13 | - |  |
| Level 4, Life-Death | .09 | .04 | -.07 | .06 | .09 | .11 | - |
| Level 4, Injury | -.02 | .02 | -.02 | .05 | -.02 | .51* | .09 |

*. Correlation is significant at the 0.01 level (2-tailed).
